# Identification of matrix metalloproteinase-2 and -9 activities within the intestinal mucosa of dogs with chronic enteropathies

**DOI:** 10.1186/s13028-018-0371-y

**Published:** 2018-03-12

**Authors:** Mohsen Hanifeh, Minna Marjaana Rajamäki, Pernilla Syrjä, Laura Mäkitalo, Susanne Kilpinen, Thomas Spillmann

**Affiliations:** 1 Department of Equine and Small Animal Medicine, Faculty of Veterinary Medicine, University of Helsinki, P.O. Box 57, (Viikintie 49), 00014 Helsinki, Finland; 2 Department of Clinical Sciences, Faculty of Veterinary Medicine, University of Tabriz, 5166616471 Tabriz, Iran; 3 Department of Veterinary Biosciences, Faculty of Veterinary Medicine, University of Helsinki, P.O. Box 66, (Agnes Sjöberginkatu 2), 00014 Helsinki, Finland; 4 Children’s Hospital, Helsinki University Central Hospital, University of Helsinki, P.O. Box 63, (Haartmaninkatu 8), 00014 Helsinki, Finland

**Keywords:** Chronic enteropathies, Dog, Matrix metalloproteinase 2, Matrix metalloproteinase 9, Zymography

## Abstract

**Background:**

Matrix metalloproteinases (MMPs) 2 and 9 are zinc- and calcium-dependent endopeptidases involved in the breakdown and reconstitution of extracellular matrix under both physiological and pathological conditions. Mucosal MMP-2 and -9 activities have been reported to be upregulated in the intestine of humans with inflammatory bowel disease (IBD), and in animal models of IBD. However, their involvement in the pathogenesis of canine chronic enteropathies (CE) is unknown. This study investigated mucosal pro- and active MMP-2 and -9 activities in dogs with CE and healthy dogs using gelatin zymography, and also to determine the association of their activities in dogs with CE with the canine IBD activity index (CIBDAI), histopathologic findings, the clinical outcome, and hypoalbuminemia. Intestinal mucosal samples from duodenum, ileum, colon, and cecum were collected from 40 dogs with CE and 18 healthy Beagle dogs.

**Results:**

In dogs with CE, the number of samples positive for mucosal pro- and active MMP-2 was significantly higher in the duodenum (P < 0.0001 and P = 0.011, respectively), ileum (P = 0.002 and P = 0.018, respectively), and colon (P < 0.0001 and P = 0.002, respectively), compared with healthy controls. Mucosal pro-MMP-9-positive samples in the duodenum and colon were significantly more frequent in dogs with CE than in healthy dogs (P = 0.0004 and P = 0.001, respectively). Despite the presence of mucosal samples positive for active MMP-9 in the intestinal segments of dogs with CE, the difference compared to healthy controls did not reach statistical significance. None of the intestinal mucosal samples in healthy dogs showed gelatinolytic activity corresponding to the control bands of active MMP-2 and -9. Mucosal active MMP-9 activities displayed a significant positive association with the severity of neutrophil infiltration in the duodenum (P = 00.040), eosinophils in the cecum (P = 00.037), and the CIBDAI score for ileum samples (P = 0.023). There was no significant association of pro- and active MMP-2 and -9 levels with the clinical outcome or hypoalbuminemia.

**Conclusions:**

This study is the first to demonstrate upregulation of mucosal pro- and active MMP-2 and pro-MMP-9 in the intestine of dogs with CE compared to healthy dogs. The results provide supporting evidence for the possible involvement of MMP-2 and -9 in the pathogenesis of canine CE.

## Background

Chronic enteropathy (CE) is a term used to describe a group of inflammatory conditions of the intestinal tract of unknown cause in dogs [1]. Canine CE causes chronic gastrointestinal signs such as vomiting, diarrhea, tenesmus, hematochezia, and decreased appetite and leads to weight loss [1, 2]. There are different clinical types of CE in dogs, which are determined by their response to treatment and defined as food-responsive diarrhea or enteropathy (FRD or FRE), antibiotic-responsive diarrhea or enteropathy (ARD or ARE), steroid-responsive diarrhea or enteropathy (SRD or SRE), or steroid-non-responsive diarrhea or enteropathy (SNRD or SNRE) [3, 4]. Synonymous for CE, the term inflammatory bowel disease (IBD) has also been used in dogs, for example in a review determining canine IBD/CE according to the response to treatment [3]. While the etiology and pathophysiology of canine CE are not fully understood, interactions between the mucosal immune system, the host genetic susceptibility, and the environment such as microbial antigens and dietary antigens, have been identified as potential causative factors in the development of chronic gastrointestinal inflammation [3, 5–8]. The occurrence of an aberrant immune response to antigens derived from endogenous microbiota is likely to play an important role in CE pathogenesis; however, the specific pathways leading to tissue injury and intestinal inflammation are not fully understood [3, 8, 9].

Matrix metalloproteinases (MMPs) are a group of zinc- and calcium-dependent endopeptidases that are important in the turnover of extracellular matrix (ECM) and cell migration [10, 11]. In addition, MMPs proteolytically activate or degrade a variety of non-matrix substrates, including chemokines, cytokines, growth factors, and junctional proteins. Therefore, they play important roles in inflammatory responses [10]. MMPs have been divided according to their domain structure and substrate specificity into gelatinases, collagenases, stromelysins, elastases, and membrane-type MMPs [12]. Gelatinases are composed of two members: MMP-2 (gelatinase A) and MMP-9 (gelatinase B), which degrade similar substrates, such as gelatin, collagen types IV and V, elastin, laminin, fibronectin, and proteoglycans [12–14]. Both MMP-2 and -9 have pro and active forms with different molecular weights. MMP-2 is primarily produced by stromal cells, including fibroblasts, myofibroblasts, and endothelial cells [15, 16]. MMP-9 is mainly produced by neutrophils and to a lesser extent by eosinophils, monocytes, macrophages, lymphocytes, and epithelial cells [14, 15, 17–19]. Intestinal mucosal MMP-2 and -9 activities have been reported to be upregulated in humans with IBD (in both Crohn’s disease [CD] and ulcerative colitis [UC]), and also in animal models of human IBD [13–15, 20, 21]. In a mouse model of colitis, MMP-2 was observed to play a protective role against tissue damage, possibly through the regulation of epithelial barrier function [10, 13, 14]. However, in humans with IBD, it contributes to ECM remodeling and the degradation of basal membrane type IV collagen, leading to intestinal ulceration, epithelial damage, and/or fistula formation [12, 15, 22–24]. In both humans with IBD and animal models of human IBD, MMP-9 plays a crucial role in both the induction of intestinal inflammation and wound healing. It promotes neutrophil migration, increases paracellular permeability, and reduces the adhesion complex integrity of the epithelium. In addition, MMP-9 interferes with re-epithelialization, resulting in impaired wound healing in cornea, skin, endothelial cells, and also in cultured intestinal epithelial cells [10, 13–15, 25].

In healthy dogs, we determined mucosal MMP-2 and -9 activities in different intestinal segments of Beagles in a previous study [26]. Only inactive pro-forms of MMP-2 and -9 were detected in the intestinal mucosa. Thus far, it has been unknown whether MMP-2 and -9 are also involved in the pathogenesis of canine CE. However, in dogs with CE, gene expression of MMP-1, -3, and -13 has been reported to be upregulated in the intestinal mucosa [27].

Therefore, the hypothesis of this study was that dogs with CE have higher MMP-2 and -9 activities in their intestinal mucosa than healthy dogs. The study also aimed at evaluating the association of intestinal mucosal MMP-2 and -9 activities with histological changes, the canine inflammatory bowel disease activity index (CIBDAI), the clinical outcome, and hypoalbuminemia in dogs with CE.

## Methods

### Animals

In this study, 52 dogs were included when undergoing routine gastroduodenoscopy and/or colonoscopy at the Veterinary Teaching Hospital, Faculty of Veterinary Medicine, University of Helsinki, Finland, due to chronic gastrointestinal (GI) signs such as vomiting, diarrhea, tenesmus, hematochezia, and/or weight loss. The study was prospectively planned and ethically approved by the Finnish National Animal Experiment Board (study license numbers: ESAVI/6973/04.10.03/2011 and ESAVI/10384/04.10.07/2014). Informed owner consent was obtained at the time the dogs were enrolled for performing gastroduodenoscopy and/or colonoscopy.

As a control group, we used stored intestinal tissue samples taken from 18 healthy laboratory Beagle dogs that underwent post-mortem examinations when finishing other unrelated studies. These studies were approved by the Finnish National Animal Experiment Board (study license numbers: ESLH-2007-09833/Ym-23, ESAVI 2010-04178/Ym-23 and ESAVI/7290/04.10.03/2012). The dogs were housed according to European Union guidelines in groups in indoor pens with access to outdoor runs. The indoor environmental temperature was maintained between 15 and 24 °C. The dogs were exposed to both natural and artificial light (from 7:00 to 16:00). They were fed a standard commercial diet and were evaluated as healthy based on history, physical examination, a complete blood count, serum biochemistry, and fecal examination. Intestinal mucosal samples were collected from the duodenum, ileum, and colon (n = 18, each, and cecum (n = 6), snap-frozen in liquid nitrogen, and stored at − 80 °C for MMP-2 and -9 determination.

For canine patients, the inclusion criteria were chronic gastrointestinal signs such as vomiting, diarrhea, tenesmus, hematochezia, and/or weight loss lasting longer than 3 weeks. Diagnostic tests were performed on each dog to exclude underlying infectious or extraintestinal disorders, which included a complete blood count, serum biochemical analysis, fecal examination for parasites, abdominal ultrasound, and gastroduodenoscopy/colonoscopy with biopsy. The diagnosis of chronic enteropathy (CE) was based on clinical, laboratory, endoscopic, and histopathologic criteria [28, 29].

### Clinical examinations of dogs with CE

The clinical severity of disease in dogs with CE was determined by the CIBDAI score at the time of entering the study and after treatment [30]. In brief, the CIBDAI score was evaluated using six prominent GI related signs (attitude and activity, appetite, vomiting, stool consistency, stool frequency, and weight loss). These were scored based on their severity from 0 to 3. The total CIBDAI score represents the summation of all individual scores and was classified as insignificant (score 0–3), mild (score 4–5), moderate (score 6–8), or severe (score ≥ 9). The clinical outcome of dogs with CE was determined based on their response to treatment and since not all included dogs developed diarrhea as a clinical sign, the type of CE was defined as FRE, ARE, SRE, and SNRE [3, 4].

### Serum albumin concentration in dogs with CE

The concentration of serum albumin was measured in each dog with CE and an albumin level of < 20 g/L was considered to indicate hypoalbuminemia. The severity of hypoalbuminemia was classified with a score of 1 (15–19.9 g/L), 2 (12–14.9 g/L), or 3 (< 12 g/L) [1].

### Histological examinations

For histologic examination of canine patients and controls, mucosal tissue samples collected from the intestine by endoscopy or necropsy were fixed in 4% formaldehyde solution in phosphate buffered saline, embedded in paraffin, sectioned (3–5 µm), and stained with hematoxylin and eosin (HE). The intestinal tissue samples were evaluated and scored by a single pathologist (PS) using the guidelines of the World Small Animal Veterinary Association (WSAVA) Gastrointestinal Standardization Group [28, 29]. The pathologist was blinded to the results of clinical and laboratory examinations, as well as the mucosal MMP-2 and -9 activities of each case. The severity of histological changes in different segments of the intestine was evaluated and scored as 0 = normal, 1 = mild, 2 = moderate, or 3 = severe according to the WSAVA standardization guidelines. The total histological change score, representing the sum of morphologic and inflammatory scores, was classified as insignificant (score 0–4), mild (score 5–9), moderate (score 10–14), severe (score 15–19), or very severe (score ≥ 20). Ileal and cecal biopsies were scored using the guidelines provided for the interpretation of duodenal and colonic biopsies due to the lack of specific templates for these intestinal parts in the guidelines of the WSAVA Gastrointestinal Standardization Group.

### Gelatin zymography

In control dogs, the intestinal mucosa was separated from the underlying muscularis layer in the snap-frozen intestinal tissue samples and stored in − 80 °C for further analysis. Snap-frozen intestinal mucosal samples from healthy dogs and dogs with CE were homogenized using Precellys 24 ceramic beads (Bertin Technologies, Paris, France) in ice-cold extraction buffer at an extraction buffer to tissue ratio of 20:1, as previously described [26]. Samples were then centrifuged and the supernatants were collected and stored at − 80 °C for MMP-2 and -9 determination. Protein concentrations of the supernatants were measured with bicinchoninic acid protein assay reagents (Pierce, Rockford, IL, USA). MMP-2 and MMP-9 activities in supernatant were measured by gelatin zymography in mini-gels as previously described in detail [26]. Briefly, supernatants were separated by electrophoresis in an 11% polyacrylamide gel impregnated with 0.7 mg/mL of gelatin as a substrate. Each lane of the gel was loaded with 20 µL of supernatant containing 10 µg of total protein mixed with a 10 µL aliquot of loading buffer. After electrophoresis, the gels were first soaked in renaturing buffer (2.5% Triton X-100) and then in zymogram developing buffer (50 mM Tris Base, pH 7.5, containing 200 mM NaCl, 5 mM CaCl_2_.2H_2_O, and 0.02% Brilj-35). They were then incubated for 18 h at 37 °C and were finally stained. Zymogram developing buffer contains divalent metal cations, which are required for enzymatic activation of both the pro and active enzymes. The areas of proteinase activity were visualized as clear bands by washing the gels with distilled water. As a control, each gel was loaded with diluted (1:600) recombinant human MMP-2 (9 μL) and -9 (8 μL) (R&D Systems, Minneapolis, MN, USA), respectively. The activities of pro- and active MMP-2 and -9 for each sample were expressed in arbitrary units (AU) related to the level of pro-MMP-2 of the positive-control standard loaded on each gel. Each band’s activity was reported as the mean of two different measurements of the same sample due to the duplication of each sample in the gel.

### Statistical analysis

Data are presented as the number (and percentage) of samples positive for MMP-2 and -9 or the median (range) of their activities, as appropriate. The differences in pro- and active MMP-2 and -9 positive samples in the duodenum, ileum, colon, and cecum between dogs with CE and healthy dogs were analyzed using Fisher’s exact test. Associations of the CIBDAI score and pro- and active MMP-2 and -9 activities were evaluated using Spearman’s correlation test. The same test was used to analyze the correlation between hypoalbuminemia and pro- and active MMP-2 and -9 activities in dogs with CE. The Kruskal–Wallis test was used to analyze the association between pro- and active MMP-2 and -9 activities and histological changes and the clinical outcome (FRE, ARE, SRE, and SNRE) in dogs with CE. P values < 0.05 were considered statistically significant. All statistical analyses were performed using SAS 9.3 statistical software (SAS Institute Inc., Cary, NC, USA).

## Results

### Demographics of dogs with CE and healthy dogs

The median age (range) of dogs with CE and healthy dogs was 5 years (1–13 years) and 10.5 (6–13), respectively. In the CE group, 15 dogs were intact males, 11 dogs were castrated males, 5 dogs were intact females, and 9 dogs were spayed females. In the healthy Beagle control group, 8 dogs were intact males and 10 dogs were intact females. The breeds of dogs with CE were mixed breed (6), Rottweiler (2), German Shepherd dog (2), Shetland Sheepdog (2), Parson Russell Terrier (2), Rough Collie (2), Standard Poodle (2), and one each of the following: Alaskan Malamute, Bichon Frise, Border Terrier, Chow Chow, Dalmatian, English Bulldog, Golden Retriever, Havanese, Irish Terrier, Jack Russell Terrier, Long-haired Dachshund, Mudi, Norwegian Lundehund, Rhodesian Ridgeback, Siberian Husky, Silky Terrier, Smooth Collie, Spanish Water Dog, Staffordshire Bull Terrier, Toy Poodle, West Highland White Terrier, and White Shepherd dog.

**Fig. 1:**
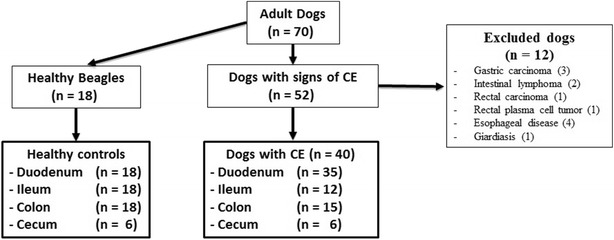
Flow diagram displaying inclusion/exclusion criteria and group distribution of all dogs enrolled in the study. *CE* chronic enteropathies

Of the 52 dogs with chronic GI signs, 12 dogs needed to be excluded from further analysis. Four dogs had primary esophageal disorders, and one dog was positive for *Giardia* sp. on fecal examination. Seven dogs had gastrointestinal neoplasia (3 gastric adenocarcinoma, 2 lymphoma, 1 rectal adenocarcinoma, and 1 rectal plasma cell tumor). Finally, 40 dogs with CE were included in the study analysis. Gastroduodenoscopy was performed in 25 dogs, gastroduodenoscopy and colonoscopy in 10 dogs, and only colonoscopy in five dogs. During the endoscopic examinations, a total of 68 intestinal mucosal biopsies were collected from four different parts of the intestine [duodenum (n = 35), ileum (n = 12), colon (n = 15), and cecum (n = 6)] (Fig. 1). The flow diagram in Fig. 1 shows the group distribution and inclusion/exclusion criteria of all dogs enrolled in the study.

### Mucosal MMP-2 and -9 activities

**Fig. 2:**
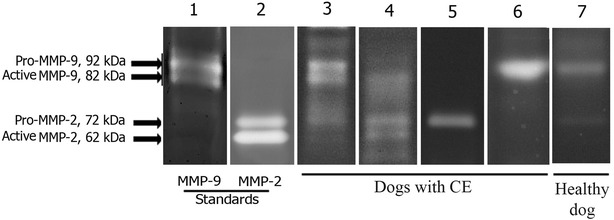
Representative zymogram from intestinal mucosa samples. Recombinant human pro- and active MMP-2 (lane 2) and -9 (lane 1), pro- and active MMP-2 and -9 positive samples in dogs with CE (lanes 3–4), pro-MMP-2 and -9 positive samples in dogs with CE (lanes 5–6), and a healthy dog (lane 7)

The zymographic analyses revealed that gelatinolytic activities in positive samples were at the same molecular weights as the positive control bands of pro- and active MMP-2 and -9, and were therefore considered to represent canine pro- and active MMP-2 and -9 (Fig. 2). The median and range of mucosal pro- and active MMP-2 and -9 activities in each intestinal segment in dogs with CE and healthy dogs are presented in Table 1.

**Table 1:** Mucosal pro- and active MMP-2 and -9 activities in dogs with CE and healthy dogs

Intestinal part	Group	Activity			
		Pro MMP-2 (AU) median (range)	Active MMP-2 (AU) median (range)	Pro MMP-9 (AU) median (range)	Active MMP-9 (AU) median (range)
Duodenum	Dogs with CE (n = 35)	0.01 (0–1.66)	0 (0–0.02)	0.06 (0–3.86)	0 (0–0.21)
	Healthy dogs (n = 18)	0 (0–0.64)	0 (0–0)	0.03 (0–3.32)	0 (0–0)
Ileum	Dogs with CE (n = 12)	0.02 (0–0.83)	0 (0–0.01)	0.06 (0–0.5)	0 (0–0.28)
	Healthy dogs (n = 18)	0 (0–0.52)	0 (0–0)	0.03 (0–0.45)	0 (0–0)
Colon	Dogs with CE (n = 15)	0.03 (0–0.55)	0 (0–0.05)	0.07 (0.02–0.92)	0 (0–0.04)
	Healthy dogs (n = 18)	0 (0–0.64)	0 (0–0)	0 (0–0.79)	0 (0–0)
Cecum	Dogs with CE (n = 6)	0.0 (0.01–1.48)	0 (0–0.01)	0.09 (0.02–0.59)	0.005 (0–0.05)
	Healthy dogs (n = 6)	0 (0–0.3)	0 (0–0)	0.18 (0–0.82)	0 (0–0)

Pro- and active MMP-2 and -9 activities were measured in the mucosal samples from duodenum, ileum, colon, and cecum of dogs with CE and healthy dogs

*AU* arbitrary units; *CE* chronic enteropathies; *Pro* pro-enzyme

**Fig. 3:**
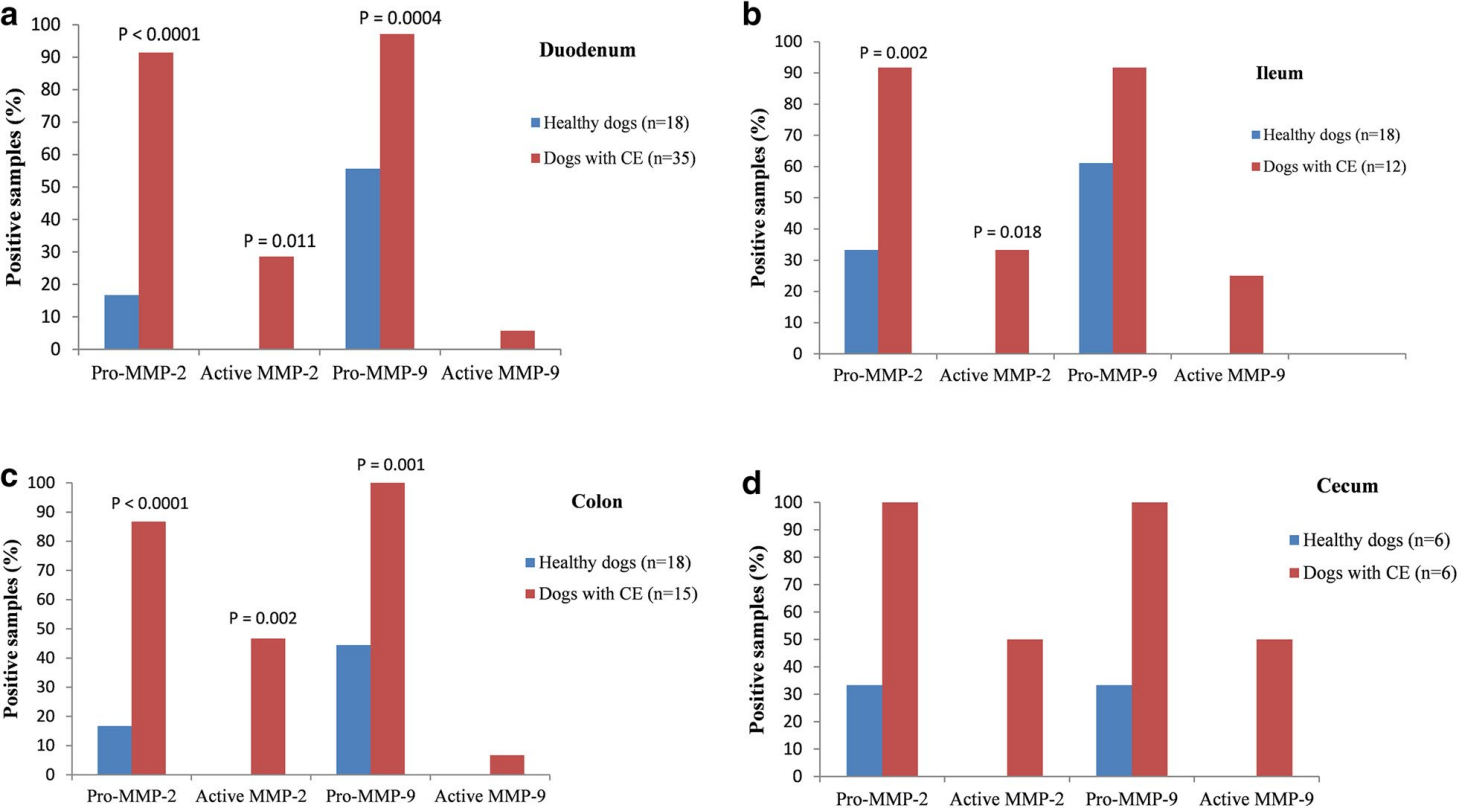
Intestinal mucosal pro- and active MMP-2 and -9 activities in CE dogs and healthy dogs. This graph shows the presence of samples positive for pro- and active MMP-2 and -9 in the mucosa of duodenum (**a**), ileum (**b**), colon (**c**), and cecum (**d**) of dogs with CE and healthy dogs. P values are based on the comparison of pro- and active MMP-2 and-9 activities in the different part of the intestine of dogs with CE versus healthy dogs

In the duodenum, dogs with CE compared to healthy dogs had a significantly higher number (and percentage) of samples positive for mucosal pro-MMP-2 (32/35 [91.4%] vs. 3/18 [16.7%]; P < 0.0001), active MMP-2 (10/35 [28.6%] vs. 0/18 [0%]; P = 0.011), and pro MMP-9 (34/35 [97.1%] vs. 10/18 [55.6%]; P = 0.0004) (Fig. 3). For active MMP-9, two positive samples were recorded in dogs with CE compared to none in healthy dogs, but this difference was not significant (Fig. 3). None of the intestinal mucosal samples in healthy dogs showed gelatinolytic activity corresponding to the control bands of active MMP-2 and -9.

In the ileum, dogs with CE compared to healthy dogs had significantly higher numbers (and percentage) of samples positive for mucosal pro-MMP-2 (11/12 [91.7%] vs. 6/18 [33.3%]; P = 0.002) and active MMP-2 (4/12 [33.3%] vs. 0/18 [0%]; P = 0.018; Fig. 3). For pro- and active MMP-9, dogs with CE had more positive samples compared to healthy dogs, but the differences were not significant (Fig. 3).

In the colon, dogs with CE compared to healthy dogs had a significantly higher number (and percentage) of samples positive for mucosal pro-MMP-2 (13/15 [86.7%] vs. 3/18 [16.7%]; P < 0.0001), active MMP-2 (7/15 [46.7%] vs. 0/18 [0%]; P = 0.002), and pro-MMP-9 (15/15 [100%] vs. 8/18 [44.4%]; P = 0.001, Fig. 3). However, in dogs with CE, only one had active MMP-9-positive samples compared to healthy dogs with none (Fig. 3).

In the cecum, despite a higher percentage of samples positive for pro- and active MMP-2 and -9 in dogs with CE compared to healthy dogs, the differences between them did not reach statistical significance (Fig. 3).

### Mucosal pro- and active MMP-2 and -9 activities and histological changes

In the ileum, pro-MMP-2 activities were significantly higher in mucosal samples with a normal epithelium (P = 0.032) and with no neutrophilic infiltration (P = 0.034). There was a significant positive association between the active MMP-2 activities and the severity of eosinophilic infiltration in cecal samples (P = 0.034). In addition, the association of active MMP-9 activities with the severity of neutrophilic infiltration in the duodenum (P = 0.040), and the severity of eosinophilic infiltration in the cecum (P = 0.037) were positive and significant. Active MMP-9 activities were also significantly higher in cecal samples with normal lamina propria lymphocyte/plasma cell scores (P = 0.049).

### Mucosal pro- and active MMP-2 and -9 activities in relation to the CIBDAI and the clinical outcome

It was only possible to record the CIBDAI score before and after treatment in 30 out of 40 dogs with CE. The CIBDAI score before and after treatment was based on either available scores taken by the clinician in charge before and after the treatment (in 13/30 and 5/30 of dogs, respectively) or retrospectively calculated by the investigators (in 17/30 and 25/30 of dogs, respectively) from information in the clinical history (before the treatment) and from telephone interviews with the owners (after the treatment). Based on the response to treatment, the outcomes of 30 dogs with CE were classified as follows: 10 dogs with FRE, 4 dogs with ARE, 13 dogs with SRE, and 3 dogs with SNRE. For pro- and active MMP-2 and -9, only active MMP-9 activities in the ileal mucosa had a strong positive correlation with the CIBDAI score before treatment in dogs with CE (*r *= 0.71, P = 0.023). In dogs with CE, we did not find a significant association between the mucosal pro- and active MMP-2 and -9 activities and the clinical outcome in each intestinal segment.

Steroid non-responsive dogs (SNRE, n = 3) either died or were euthanized due to severe clinical signs. When comparing duodenal samples, the median (range) of the CIBDAI score before treatment was higher in SNRE dogs (n = 3) than in SRE dogs (n = 12) (7 [4–7] vs. 4.5 [0–7]). Hypoalbuminemia (< 20 g/L) was present in one SNRE and two SRE dogs. The median (range) of the total histological change score was slightly higher for SNRE dogs than SRE dogs (7 [5–7] vs. 6 [1–11]). The median (range) of the pro- and active MMP-2 and -9 activities in the duodenal mucosa of SNRE dogs compared to SRE dogs were as follows: pro-MMP-2 (0.01 [0–0.02] AU vs. 0.01 [0–1.66] AU), active MMP-2 (0 [0–0.01] AU vs. 0 [0–0.01] AU), pro-MMP-9 (0.07 [0.02–0.16] AU vs. 0.09 [0–3.86] AU), and active MMP-9 (0 [0–0.21] AU vs. 0 [0–0] AU. The number of SNRE dogs was too low for statistical analysis other than descriptive comparison.

### Mucosal pro- and active MMP-2 and-9 activities in relation to hypoalbuminemia in dogs with CE

In this study, 36 dogs out of 40 (90%) had a serum albumin level of > 20 g/L, with a median (range) of 32.65 g/L (24.9–39 g/L). A total of 4 out of 40 dogs (10%) had serum albumin concentrations of < 20 g/L (median 12.2 g/L; range 11–13 g/L), with a hypoalbuminemia severity scores of 2 for two dogs and of 3 for another two [1]. Duodenal biopsy samples were taken from the four hypoalbuminemic CE dogs, and the correlations between mucosal pro- and active MMP-2 and-9 activities and hypoalbuminemia were evaluated. However, there was no significant correlation between pro-MMP-2 or active MMP-9 activities in the duodenal mucosa and hypoalbuminemia in dogs with CE. In hypoalbuminemic dogs with CE, compared to normoalbuminemic dogs with CE, the number (percentage) of positive samples in the duodenal mucosal was as follows: pro-MMP-2 (3/4 [75%] vs. 29/31 [93.5%]), active MMP-2 (1/4 [25%] vs. 9/31 [29%], pro-MMP-9 (3/4 [75%] vs. 31/31 [100%]), and active MMP-9 (1/4 [25%] vs. 1/31 [3.2%]). The median (range) of the pro- and active MMP-2 and -9 activities in the duodenal mucosa of hypoalbuminemic dogs with CE compared to normoalbuminemic dogs with CE was as follows: pro-MMP-2 (0.01 [0–0.017] AU vs. 0.01 [0–1.66] AU), active MMP-2 (0 [0–0.01] AU vs. 0 [0–0.02] AU), pro-MMP-9 (0.14 [0–0.74] AU vs. 0.05 [0.01–3.86] AU), and active MMP-9 (0 [0–0.21] AU vs. 0 [0–0.1] AU.

## Discussion

This study is the first to report mucosal MMP-2 and -9 activities in the intestine of dogs with CE. The number of samples positive for pro- and active MMP-2 and pro-MMP-9 was higher in the mucosa of dogs with CE compared to healthy dogs in all intestinal segments when determined using gelatin zymography. Similar findings were also reported for active MMP-2 and pro-MMP-9 in the colonic mucosa of humans with IBD compared to healthy controls [20]. A significantly higher percentage of colonic mucosal samples in dogs with CE had pro-MMP-2 activity compared to healthy dogs (86.7% vs. 16.7%). However, the percentage of samples with colonic mucosal pro-MMP-2 activity was the same in human patients with IBD and healthy controls, and activity was detected in 80% of the samples [20]. Active MMP-2 was detected in 46.7% of the colonic samples of dogs with CE. However, no activity was found in colonic mucosal samples of healthy dogs. Similarly to our findings, Baugh et al. [20] did not detect active MMP-2 activities in the colonic mucosa of healthy humans. However, they found active MMP-2 activities in 35% of samples in humans with IBD. Under normal conditions, MMP-2 is believed to participate in the maintenance of collagen homeostasis and intestinal tissue remodeling. In human IBD, MMP-2 has been reported to contribute to ECM remodeling and the degradation of basal membrane type IV collagen, leading to intestinal ulceration, epithelial damage, and/or fistula formation [12, 15, 22–24]. It appears that intestinal tissue turnover is increased during intestinal inflammation, tissue destruction, and healing processes, and demands greater MMP-2 activities in human IBD [15]. In contrast, it has been reported that MMP-2 plays a protective role against tissue damage, possibly through the regulation of epithelial barrier function, in an MMP-2 knockout mouse model of IBD [10, 13, 14]. In our study, it appears that having higher activities of pro- and active MMP-2 in dogs with CE indicates the possible involvement of this enzyme in the pathogenesis of canine chronic enteropathies. In addition, we showed that the samples with higher pro-MMP-2 activities have an association with a normal epithelium and lamina propria without neutrophils in the ileum. Similarly to the study on an MMP-2 knockout mouse model of IBD, it is possible that MMP-2 has a protective role against tissue damage through the regulation of epithelial barrier function in dogs; however, more research is needed to clarify the role of MMP-2 in canine CE.

Mucosal pro-MMP-9 was detected in more than 90% of the samples in the intestinal segments of dogs with CE, while this form of enzyme was detected in 33–61% of the intestinal mucosal samples of healthy dogs. Therefore, pro-MMP-9 activities are elevated in dogs with CE compared to healthy dogs, which is similar to the findings of Baugh et al. [20] in humans with IBD. In our study, we did not detect the active form of MMP-9 in the mucosa of healthy dogs. Similar findings were also reported in the colonic mucosa of healthy humans [20]. In dogs with CE, the active form of MMP-9 was detected in 5.7, 25, 5.6, and 50% of duodenal, ileal, colonic, and cecal samples, respectively. In the duodenal and cecal samples, we also found a positive and significant association between active MMP-9 activities and the severity of lamina propria neutrophilic and eosinophilic infiltration, with these cells being proposed as sources of MMP-9. In comparison to human IBD, the percentage of active MMP-9-positive samples in the colon of dogs with CE was much lower (55% vs. 5.6%) [20]. This difference could be due to the different pathophysiology of canine and human chronic colitis. A higher number of infiltrated neutrophils and more ulcerative lesions in humans with chronic colitis could be the possible reasons for the higher active MMP-9 activities in humans [15]. In future studies, immunohistochemistry should also be included to assess the localization of MMP-2 and -9 in the intestinal mucosa and their correlation with intestinal pathologies in dogs.

We found a positive correlation between active MMP-9 activities in the ileal mucosa and the CIBDAI score before treatment in dogs with CE, which is similar to the relationship between mucosal MMP-9 and the disease activity index in a rat model of colitis [31], and between fecal MMP-9 and clinical activities of ulcerative colitis in humans [32]. However, due to the low number of active MMP-9-positive samples (n = 3) in the ileal samples of dogs with CE in our study, the results should not be over-interpreted.

The association between an aberrant intestinal expression of MMP-2 and -9 and human IBD is now well established [33]. MMP-2 and -9 have been investigated as biomarkers and diagnostic tool for human IBD. The level of fecal MMP-9 in UC patients correlates with disease activity and has recently been proposed as a biomarker of the disease. By measuring fecal MMP-9 levels, it was possible to distinguish UC from diarrhea predominant irritable bowel syndrome with 85% sensitivity and 100% specificity [34]. Serum level of MMP-9 have also been shown as a potential tool in the prediction of CD activity status in children [35]. In addition, MMP-2 and MMP-9 levels in urine of pediatric patients with IBD have been reported as useful novel non-invasive biomarkers to predict CD and UC independently in children [33]. Our study is the first to demonstrate an upregulation of mucosal pro- and active MMP-2 and pro-MMP-9 in the intestine of dogs with CE. This is a promising indication that MMPs play a role in canine CE with the potential of being used as biomarkers of active disease and disease severity. In the present study, however, we did not find a significant association between mucosal MMP-2 and -9 activities and the type of clinical outcome in dogs with CE which could be due to the low number of dogs with a certain clinical outcome (especially SNRE dogs, n = 3). In addition, the comparison between intestinal segments led to rather low case numbers when assessing the association between mucosal MMP-2 and -9 activities with the type of clinical outcome. Further studies with sufficient number of canine patients in each types of CE is needed to evaluate the relationship between MMP-2 and -9 activities and canine CE subtypes. MMP expression in the intestine or elsewhere in the body, e.g. serum or feces, may in the future help in properly differentiating disease subtypes and severity, and to enable tailored treatment choices for individuals.

A unique therapeutic option for human IBD is targeting MMPs which has been investigated in animal models of IBD. Inhibition of MMPs with non-selective inhibitors (e.g. Marimastat, Batimastat) has been shown to reduce mucosal damage and colitis induced by dextran sodium sulfate (DSS) in animal models [33]. However, the development of MMP inhibitors in humans has been limited by their poor selectivity [33]. To target MMPs, monoclonal antibodies against MMP-2 and MMP-9 have been developed and tested on murine models of IBD. These antibodies were significantly reducing the severity of the DSS induced colitis in mice [33, 36]. However, due to the myriad roles of MMPs in vivo and their ubiquitous expression throughout the body, using monoclonal antibodies remains a serious concern and a barrier to their current therapeutic use in human IBD [33]. Whether targeting MMPs using monoclonal antibodies in canine CE patients is beneficial for CE treatment is not known yet and needs to be studied.

It has been shown that treatment of human IBD with immunosuppressive medications could reduce the levels of MMPs in the intestinal mucosa and serum. Makitalo et al. [37] showed in people that the treatment of CD patients with anti-TNF-α therapy (infliximab or adalimumab) or with corticosteroids and other immunosuppressive drugs (methotrexate or azathioprine) decreased stromal MMP-9 and epithelial MMP-7 as assessed by immunohistochemistry methods. However, there are different reports regarding the response of MMP-9 to therapy in serum. Gao et al. [38] reported that serum levels of MMP-9 decrease in adults in response to infliximab therapy; however, Makitalo et al. [39] reported no significant changes in serum MMP-9 levels after therapy. In mice, mangiferin, a bioactive compound of the mango, attenuated DSS induced colitis through directly reducing the activity of mucosal TNF-α and MMP-9 [40]. Future studies in CE dogs should be planned as treatment follow up studies with repeated biopsies to evaluate the treatment effects of different medication especially immunosuppressive drugs such as prednisolone on the intestinal mucosal MMP-2 and -9 activities.

We did not find significant correlations between pro- and active MMP-2 and -9 activities in the duodenal mucosa and hypoalbuminemia in dogs with CE. Since only 10% (4/40) of the dogs had hypoalbuminemia (< 20 g/L), the number of samples was too low for an appropriate power of analysis and it could be the reason why we did not find significant correlations between MMP2 and 9 activities. In humans, to our knowledge, there has been so far no report studying MMP-2 and -9 activities in patients with protein losing enteropathies. In future prospective studies, the necessary number of cases of CE dogs with hypoalbuminemia should be prospectively estimated by using power analysis, for which the data of the current study are helpful. In the future, it will also be necessary to focus more on advanced cases with intestinal protein loss, a patient group that was underrepresented in this study and also looking for other MMPs. Another limitation of our study is that the cobalamin and folate concentrations were not measured in a sufficient number of dogs with CE to allow statistical analysis. Therefore, future prospective studies need to include a variety of laboratory data to assess possible associations/correlations.

## Conclusions

This is the first study to demonstrate that mucosal pro- and active MMP-2 and pro-MMP-9 are upregulated in the intestine of dogs with CE compared to healthy dogs. Compared to humans with IBD, the active form of MMP-9 has been detected in a rather small number of canine patients, which could be due to the low number of granulocytes found in the intestinal mucosa of dogs with CE. The results provide supporting evidence for the possible involvement of MMP-2 and -9 in the pathogenesis of canine CE. Further research is needed to assess the localization of MMP-2 and -9 in the canine intestinal mucosa, their presence and activity level in advanced CE with intestinal protein loss, and their relationship with other inflammatory markers in canine chronic enteropathies. Furthermore, their activities in fecal samples should be determined to examine their possible usefulness as non-invasive biomarkers of intestinal inflammation.

## Abbreviations

ARD: antibiotic-responsive diarrhea
ARE: antibiotic-responsive enteropathy
AU: arbitrary unit
CD: Crohn’s disease
CE: chronic enteropathy
CIBDAI: canine inflammatory bowel disease activity index
cTLI: canine trypsin-like immunoreactivity
DSS: dextran sodium sulfate
ECM: extracellular matrix
FRD: food-responsive diarrhea
FRE: food-responsive enteropathy
GI: gastrointestinal
HE: hematoxylin and eosin
IBD: inflammatory bowel disease
MMPs: matrix metalloproteinases
SRD: steroid-responsive diarrhea
SRE: steroid-responsive enteropathy
SNRD: steroid non-responsive diarrhea
SNRE: steroid non-responsive enteropathy
TNF-α: tumor necrosis factor-alpha
UC: ulcerative colitis
WSAVA: World Small Animal Veterinary Association
